# The miR528-*D3* Module Regulates Plant Height in Rice by Modulating the Gibberellin and Abscisic Acid Metabolisms

**DOI:** 10.1186/s12284-022-00575-3

**Published:** 2022-05-20

**Authors:** Juan Zhao, Xing Liu, Mei Wang, Lingjuan Xie, Zhengxin Wu, Jiuming Yu, Yuchen Wang, Zhiqiao Zhang, Yufang Jia, Qingpo Liu

**Affiliations:** The Key Laboratory for Quality Improvement of Agricultural Products of Zhejiang Province, College of Advanced Agricultural Sciences, Zhejiang A&F University, Lin’an Hangzhou, 311300 People’s Republic of China

**Keywords:** *Oryza sativa* L., Dwarf, Plant height, Gibberellin, Abscisic acid, Strigolactone

## Abstract

Plant height, as one of the important agronomic traits of rice, is closely related to yield. In recent years, plant height-related genes have been characterized and identified, among which the *DWARF3 (D3)* gene is one of the target genes of miR528, and regulates rice plant height and tillering mainly by affecting strigolactone (SL) signal transduction. However, it remains unknown whether the miR528 and *D3* interaction functions in controlling plant height, and the underlying regulatory mechanism in rice. In this study, we found that the plant height, internode length, and cell length of internodes of *d3* mutants and miR528-overexpressing (OE-miR528) lines were greatly shorter than WT, *D3*-overexpressing (OE-D3), and miR528 target mimicry (OE-MIM528) transgenic plants. Knockout of *D3* gene (*d3* mutants) or miR528-overexpressing (OE-miR528) triggers a substantial reduction of gibberellin (GA) content, but a significant increase of abscisic acid (ABA) accumulation than in WT. The *d3* and OE-miR528 transgenic plants were much more sensitive to GA, but less sensitive to ABA than WT. Moreover, the expression level of GA biosynthesis-related key genes, including *OsCPS1*, *OsCPS2, OsKO2* and *OsKAO* was remarkably higher in OE-D3 plants, while the *NECD2* expression, a key gene involved in ABA biosynthesis, was significantly higher in *d3* mutants than in WT and OE-D3 plants. The results indicate that the miR528-*D3* module negatively regulates plant height in rice by modulating the GA and ABA homeostasis, thereby further affecting the elongation of internodes, and resulting in lower plant height, which adds a new regulatory role to the *D3*-mediated plant height controlling in rice.

**Supplementary Information:**

The online version contains supplementary material available at 10.1186/s12284-022-00575-3.

## Background

As one of the important components of plant architecture, plant height is closely related to the photosynthetic efficiency and lodging resistance, and directly affects the biomass of plants in rice (Zhang et al. 2014). Accordingly, appropriate dwarfing of plant height will benefit in improving the lodging resistance of rice. In the 1950s, the discovery and application of the rice semi-dwarf gene *sd1* greatly alleviate the food shortages in at least nineteen developing countries (Monna et al. 2002; Wang and Wang 2017). It is thus feasible that genetically manipulating of functional genes should be effective in controlling plant height and further grain yield in rice.

In recent years, amounts of genes related to plant height have been characterized and identified in rice, where most of them are involved in plant hormone metabolism and signal transduction, such as gibberellin (GA), brassinolide (BR), strigolactone (SL) and abscisic acid (ABA) (Zou et al. 2019; Zhang et al. 2020a). The *OsKO2* gene encodes a kaurene oxidase that catalyzes the early steps of GA biosynthesis. The expression of *OsKO2* was repressed by LRK1 and activated by OsbZIP58, thereby affecting GA biosynthesis and internode elongation in rice (Yang et al. 2013; Wu et al. 2014). *GID1* encodes a soluble GA signal receptor, which mediates GA signal transduction and plays a major role in regulation of rice plant height (Ueguchi-Tanaka et al. 2005). Except for GA, BRs are also determinant in controlling plant height. The plant height of *BR-deficient dwarf* mutant *brd2* was about 70% of the wild type (WT) at the seedling stage, but the mutants were severe dwarfing, only 40% of the WT plants, and most of the internodes did not elongate except the uppermost internode at the mature stage (Hong et al. 2005). In addition, *D2*, *D11*/*CYP724B1*, *D61*/*OsBRI1*, *OsDWARF4* also control plant height by regulating BR synthesis and signaling (Zhang et al. 2016; Tong et al. 2018). Interestingly, previous studies reveal that the abnormal signal pathways related to SL, such as *d88*/*d14*, *d27*, *d3*, *dit1*, often cause changes in tiller number and plant height (Ishikawa et al. 2005; Arite et al. 2009; Gao et al. 2009; Lin et al. 2009). Another kind of hormone ABA negatively regulates plant growth, and results in dwarfing of plants, which often functions in the way with GA antagonism (Wang et al. 2020a, b).

In rice, *D3* encodes a leucine-rich repeat F-box protein, which is homologous to MAX2/ORE9 that controls the activity of axillary buds by participating in an SCF complex and SL signal transduction in *Arabidopsis thaliana* (Ishikawa et al. 2005; Zhao et al. 2014; Sun et al. 2018). The rice *d3* mutant exhibits a shorter plant height and increased tiller numbers (Ishikawa et al. 2005; Zhao et al. 2014; Sun et al. 2018). Recently, D3 was found to be involved in a karrikin signaling complex (D14L-D3-OsSMAX1) that mediates the regulation of rice mesocotyl elongation in the dark (Choi et al. 2020; Zheng et al. 2020). In addition, *D3* was experimentally validated to be a target gene of rice miR528 through degradome sequencing (Li et al. 2010; Zhou et al. 2010). As known, miR528 plays multifaceted roles in regulation of plant growth and development, and biotic and abiotic stress responses (Liu et al. 2015; Wu et al. 2017; Tang and Thompson 2019; Yang et al. 2019; Zhang et al. 2020b; Wang et al. 2021). However, whether the miR528 and *D3* interaction plays a role in regulation of plant height in rice, and its further underlying regulatory mechanism remains unclear.

In this study, we aim to uncover the regulatory role of the miR528-*D3* module in controlling plant height by regulating the expression of some key genes related to GA and ABA biosynthesis, leading to the changes of the content of endogenous hormones and the cell elongation in rice. This research is significant for further understanding the *D3*-mediated mechanism in regulation of plant height in plants.

## Results

### miR528 and *D3* Exhibit Opposite Expression Patterns Along Developmental Stages

**Fig. 1:**
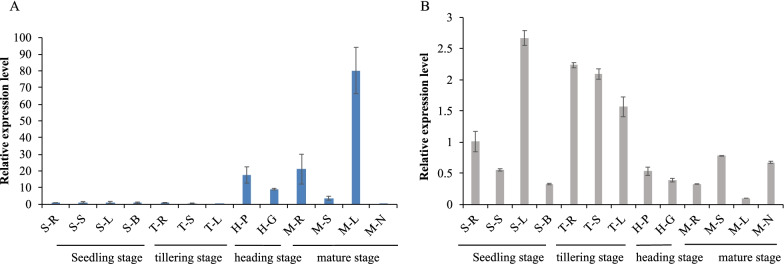
Analysis of the miR528 and *D3* genes expression pattern in rice. **A** Expression pattern analysis of miR528 in various tissues by qRT-PCR. **B** Expression pattern analysis of D3 in various tissues by qRT-PCR. S-R, root at the 7-day seedlings; S-S, stem at the 7-day seedlings; S-L, leaf at the 7-day seedlings; S-B, stem base at the 7-day seedlings; T-R, root at the tillering stage; T-S, stem at the tillering stage; T-L leaf at the tillering stage; H-P, Panicles at the heading stage; H-G, glume at the heading stage; M-R, root at the mature stage; M-S, stem at the mature stage; M-L, leaf at the mature stage; M-N, stem node at the mature stage. Data are shown as the means ± S.D. (n = 3)

To explore whether the *D3* expression is regulated by miR528, we first investigated their temporal and spatial expression patterns in different tissues along developmental stages. It is obvious that miR528 and *D3* showed totally different expression patterns (Fig. 1). The transcriptional level of miR528 was relatively lower in roots, shoots, and leaves at the seedling and tillering stages, but was significantly higher in the examined tissues at the heading and mature stages, except for in the stems and stem nodes at the maturity stage (Fig. 1A). On the contrary, the *D3* gene was highly expressed in roots and leaves at the seedling stage and in roots, shoots, and leaves at the tillering stage, while its expression level was greatly lower in other tested tissues, especially in leaves at the mature stage (Fig. 1B). Furthermore, the transcriptional level of *D3* gene was separately examined in the miR528-overexpressing (OE-miR528) and miR528 target mimicry (OE-MIM528) transgenic lines. The *D3* expression was significantly down-regulated in OE-miR528, but strongly up-regulated in OE-MIM528 plants (Additional file 1: Fig. S1A). These observations strongly support that the expression of *D3* is truly controlled by miR528 in rice.

### The miR528-*D3* Module Negatively Regulates Plant Height in Rice

In order to better understand whether the miR528-*D3* module have potential roles in regulation of plant height in rice, two independent *D3*-overexpressing transgenic lines (OE-D3-2 and OE-D3-9) and two homozygous *D3-*knockout mutant lines (*d3-1* and *d3-3*) were developed. The *D3* gene was significantly up-regulated in the OE-D3 lines, whose transcriptional level was about 12- and 166-fold higher than in WT (Additional file 1: Fig. S1B). The *d3-1* mutant had a deletion of three nucleotides (ACG) at the position of 101–103 bp in the coding region, leading to the deletion of the aspartic acid (D) at the 34^th^ residues, while the *d3-3* mutant contained a deletion of four nucleotides (GACG) at the position of 100–103 bp in the coding region, resulting in a frameshift mutation (Additional file 1: Fig. S2).

**Fig. 2:**
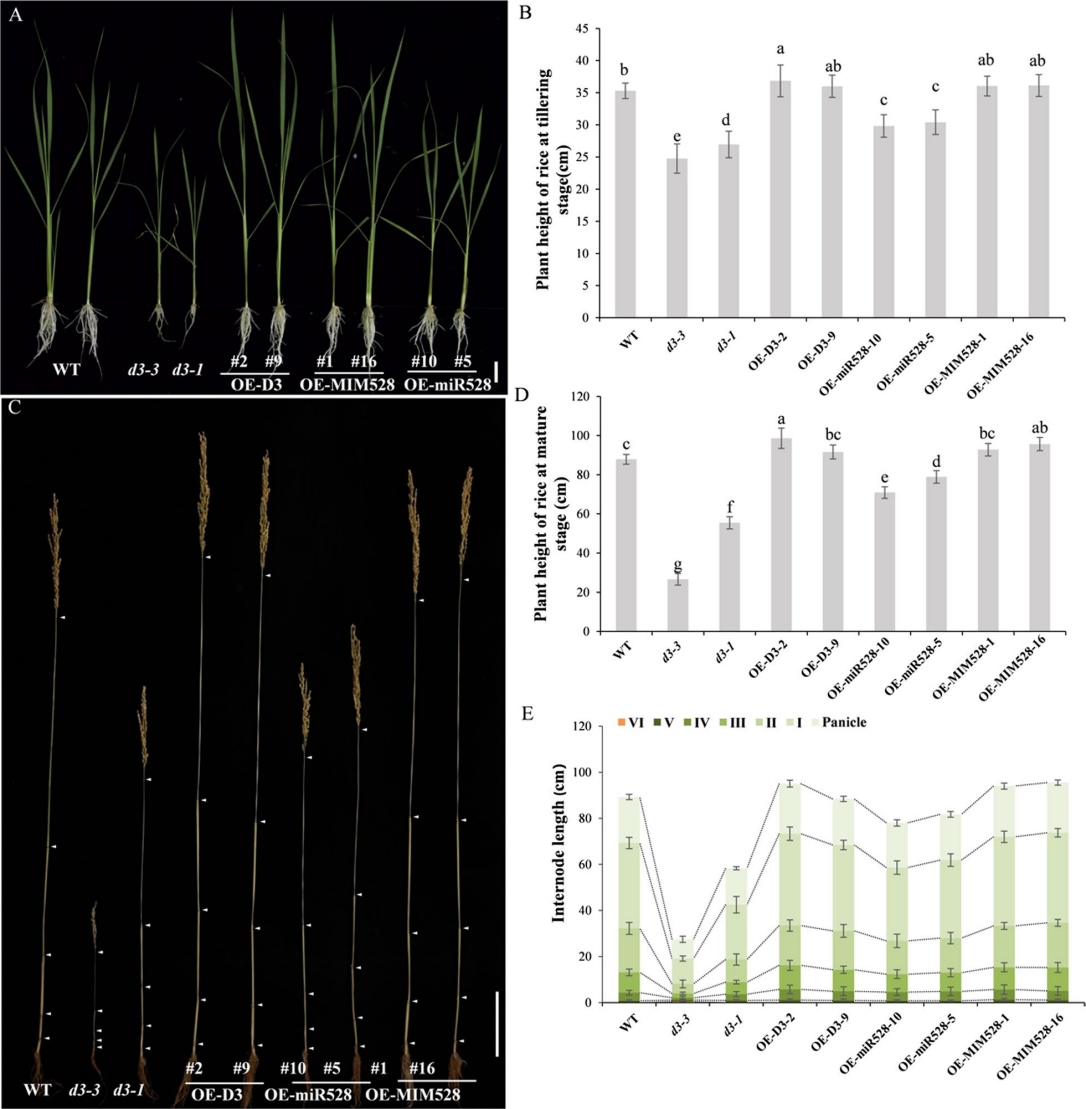
Comparison of plant height and internode length between WT and transgenic lines at the tillering and maturity stages. **A** Phenotypes of WT and transgenic lines at tillering stage. Bars, 2 cm. **B** Comparison of plant height at tillering stage. Data are shown as the means ± S.D. (n = 10). Significant differences (*P* < 0.05) are indicated by different letters. **C** Internode morphology of WT and transgenic lines. Bars, 10 cm. **D** Comparison of plant height at maturity stage. Data are shown as the means ± S.D. (n = 10). Significant differences (*P* < 0.05) are indicated by different letters. **E** Comparison of internode length of WT and transgenic lines. Data are shown as the means ± S.D. (n = 10)

Compared with WT plants, the plant height of *d3-3* and *d3-1* decreased by 29.9% and 26.4% at the tillering stage; Consistently, the plant height of OE-miR528 lines was also apparently shorter, which decreased by 15.5% and 13.8% in OE-miR528-10 and OE-miR528-5 plants. By contrast, OE-D3 and OE-MIM528 lines were slightly higher than WT (4.4% and 2.0% for OE-D3-2 and OE-D3-9, and 2.1% and 2.3% for OE-MIM528-1 and OE-MIM528-16) (Fig. 2A, B). At the maturity stage, the difference on plant height was more pronounced among transgenic lines. The plant height of *d3* mutants and OE-miR528 lines was decreased by 69.7% (*d3-3*), 36.9% (*d3-1*), 19.3% (OE-miR528-10), and 10.2% (OE-miR528-5), while the plant height of OE-D3 and OE-MIM528 lines was increased by 12.3% (OE-D3-2), 4.3% (OE-D3-9), 5.6% (OE-MIM528-1), and 8.9% (OE-MIM528-16), in comparison with WT plants (Fig. 2C, D and Additional file 1: Fig. S3). The results indicate that miR528 and *D3* genes both have regulatory effect on plant height in rice, but they function in opposite ways. In addition, *d3-3* was more dwarfed than *d3-1* at the mature stage (Fig. 2C, D and Additional file 1: Fig. S3), which may be due to the loss-of-function mutation occurred in *d3-3* plants.

### The Dwarfness of *d3* and OE-miR528 Plants are Due to the Shorter Internodes and Cell Length

To reveal the histological mechanism of the changes in plant height, we counted and measured the internode number and length of WT and transgenic lines at the maturity stage. There was no difference in internode numbers between WT and transgenic plants, both of them have five internodes (Fig. 2C). However, the internode length of *d3* and OE-miR528 lines were significantly shorter, while the internode length of OE-D3 and OE-MIM528 lines were slightly longer than WT (Fig. 2C, E). The uppermost internode exhibited the most prominent difference among different transgenics (Fig. 2C, E). Thus, it is reasonable to infer that the miR528-*D3* module affects rice plant height by controlling the length of internodes, especially the uppermost internode.

**Fig. 3:**
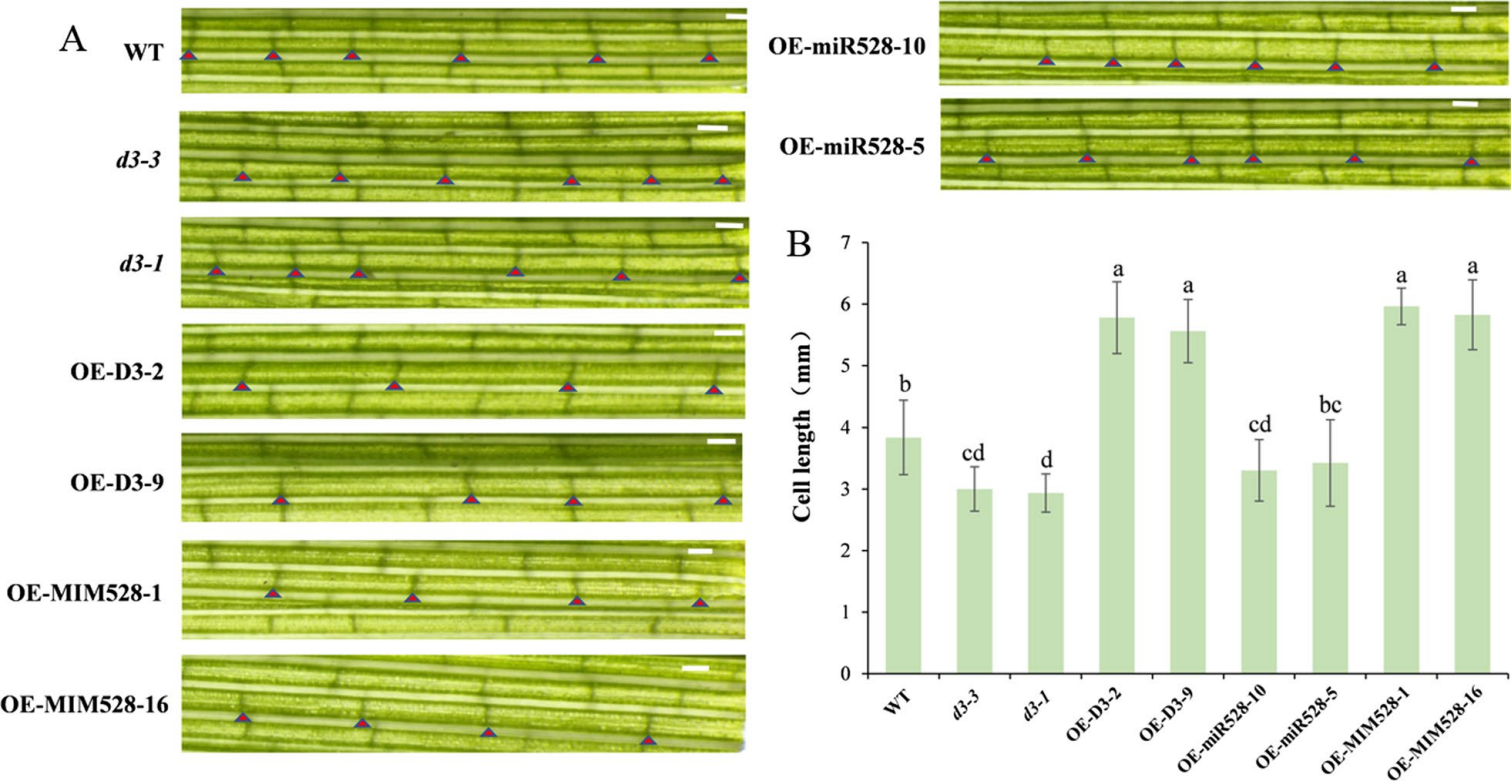
Phenotypic analysis of the internode length of WT and transgenic lines. **A** Longitudinal section of the uppermost internode from WT and transgenic lines at the tillering stage. **B** Statistical analysis of the cell length of internodes in WT and transgenic plants. Data are shown as the means ± S.D. (n = 10). Bars, 1 mm. Significant differences (*P* < 0.05) are indicated by different letters

Previous studies show that the length of internodes is related to the stem elongation and growth caused by cell division and expansion of the apical meristem (SAM) and the intermediate meristem (IM) in rice (Wang et al. 2018). Accordingly, we speculate that the observed difference in the internode length of transgenic rice plants may be due to the change in the size of cells. To verify this, longitudinal tissue sections were made from the uppermost internode of WT and transgenic lines at the tillering stage. The results showed that the cell length of *d3* and OE-miR528 lines was greatly shorter, while the cell length of OE-D3 and OE-MIM528 was significantly longer than WT (Fig. 3), indicating that the elongation of cells is responsible for the length of internodes.

### The Accumulation of GA and ABA is Severely Affected in *d3* and OE-miR528 Lines

**Fig. 4:**
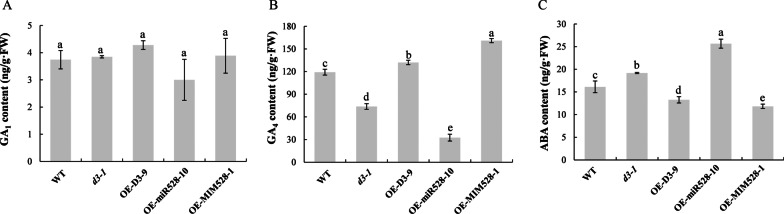
Comparative analysis of endogenous GA_1_ (**A**), GA_4_ (**B**), and ABA content (**C**) in 31-day-old seedlings of WT and transgenic lines. Significant differences (*P* < 0.05) are indicated by different letters

A variety of plant hormones affect the elongation of stems in rice, of which GA deficiency or abnormal signal transduction will cause the dwarfness of plants, while ABA has antagonistic effect of GA on plant height (Ayano et al. 2015). To clarify whether the miR528-*D3* module controls internode elongation by affecting the metabolism of GA and ABA, we determined the content of endogenous GA_1_, GA_4_ and ABA of WT and transgenic lines at the seedling stage. There was no clear difference in GA_1_ accumulation between WT and transgenic lines (Fig. 4A). However, compared with WT, *d3-1* and OE-miR528-10 lines had significantly lower GA_4_ content, whereas OE-D3-9 and OE-MIM528-1 lines contained extensively much more GA_4_ (Fig. 4B). The ABA content of *d3-1* and OE-miR528-10 lines was significantly higher than WT, while there was clearly lower ABA content in OE-D3-9 and OE-MIM528-1 lines in comparison with WT plants (Fig. 4C). Thus, the dwarfness of *d3* and OE-miR528 plants should be attributed to the highly increased endogenous ABA content and the decreased GA content.

**Fig. 5:**
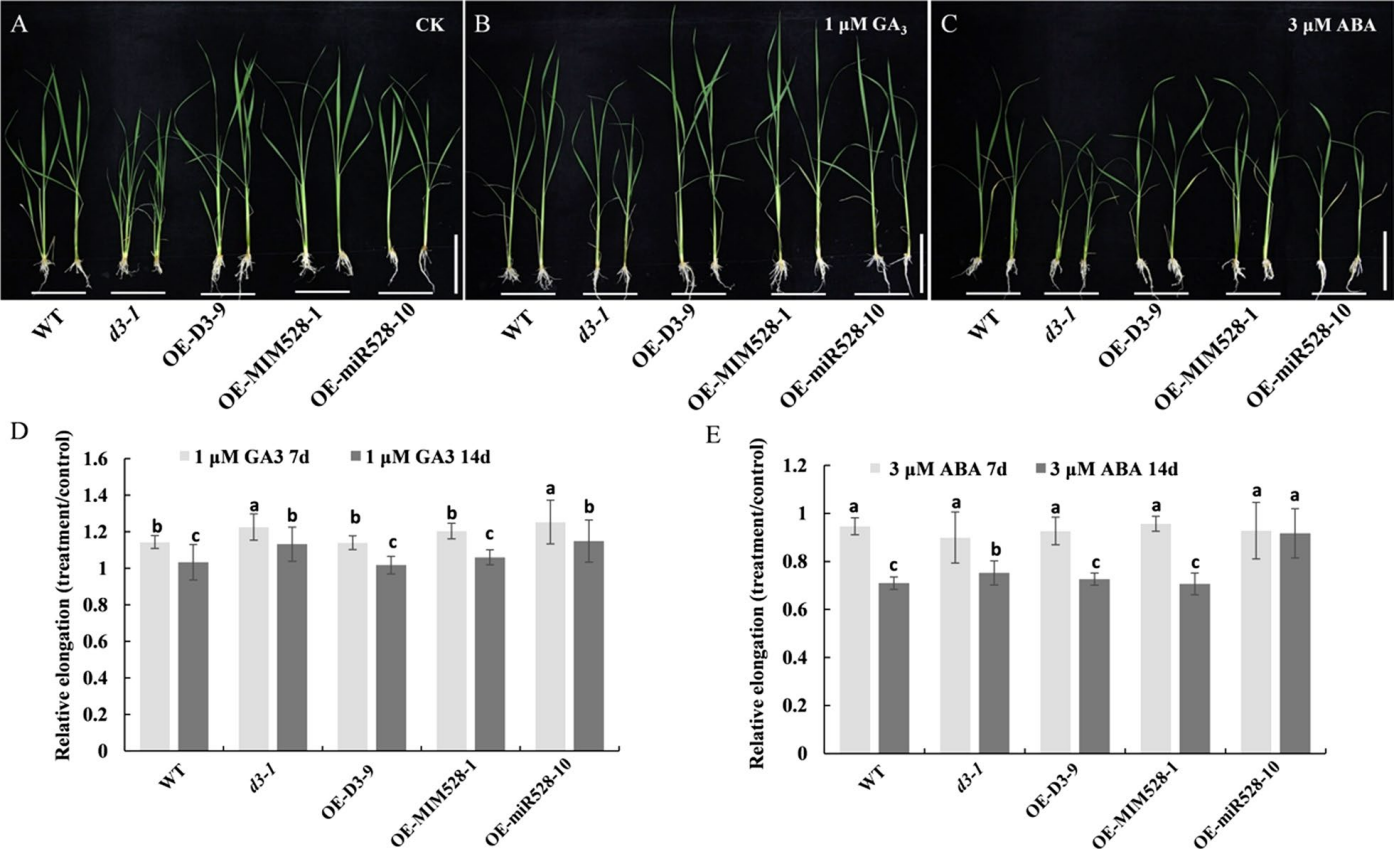
Response of WT and transgenic lines to exogenous GA and ABA treatment. **A** Phenotype of WT and transgenic plants under normal conditions; **B**, **C** Phenotypes of 14-day-old seedlings of WT and transgenic lines under the treatment with exogenous 1 μM GA_3_ (**B**) and 3 μM ABA (**C**) for 7 days. **D** Relative elongation of plant height after 1 μM GA_3_ treatment for 7 and 14 days. **E** Relative elongation of plant height after 3 μM ABA treatment for 7 and 14 days. Data are shown as the means ± S.D. (n = 10). Significant differences (*P* < 0.05) are indicated by different letters

To further verify the responses of rice plants to GA and ABA, the WT and transgenic lines were treated with exogenous GA_3_ (1 µM) and ABA (3 µM). After 7 d and 14 d of GA_3_ treatment, the plant height of *d3-1* and OE-miR528-10 increased significantly (Fig. 5A, B, D), as reflected by their significantly higher relative elongation of plant height than WT, OE-D3-9, and OE-MIM528-1 lines (Fig. 5D). However, the relative elongation of plant height of OE-D3-9 and OE-MIM528-1 lines is not significantly different from WT plants (Fig. 5D). This observation indicates that the *d3* and OE-miR528 lines are more sensitive to external GA_3_. Moreover, after 7-d of ABA supplementation, the relative elongation of plant height was not apparently different from WT and transgenic lines (Fig. 5C, E); But, along with the treatment time (14 d), the *d3-1* and OE-miR528-10 plants grew significantly much faster than WT, while OE-D3-9 and OE-MIM528-1 lines were not obviously different from WT (Fig. 5C, E), suggesting that the *d3* and OE-miR528 lines were not sensitive to the exogenous application of ABA. These results indicated that the miR528-*D3* module should be involved in the anabolic processes of GA and ABA.

### miR528-*D3* Regulates the Expression of GA and ABA Biosynthesis- and Metabolism-Related Genes

**Fig. 6:**
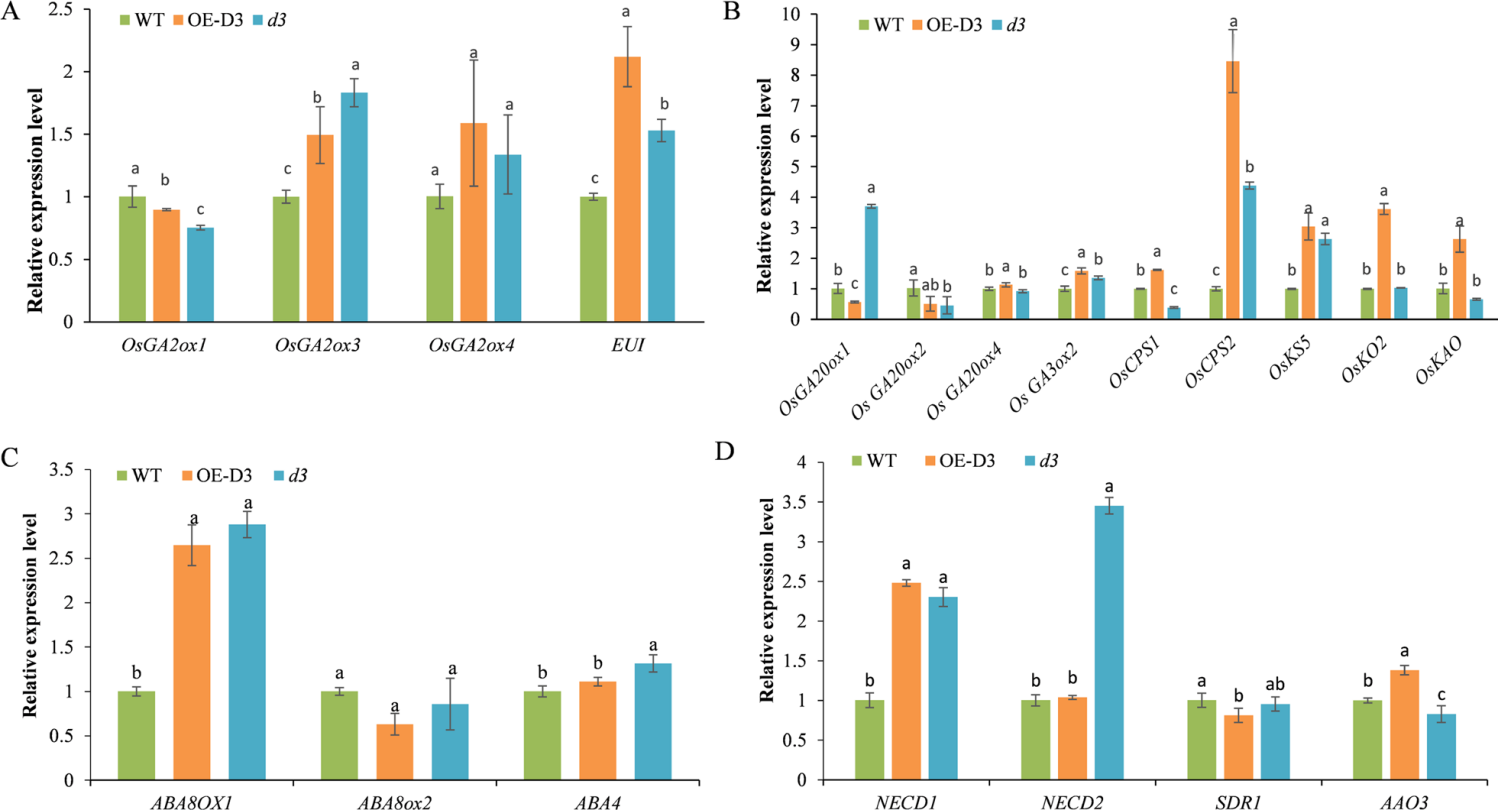
Expression analysis of some key genes involved in GA and ABA degradation and biosynthesis in WT, OE-D3 and *d3* lines. **A** Expression analysis of GA degradation-related genes. **B** Expression analysis of GA biosynthesis-related genes. **C** Expression analysis of ABA degradation-related genes. **D** Expression analysis of ABA biosynthesis-related genes. Data are presented as the means ± S.D. of three biological replicates. The *Ubquitin* gene was used as an internal control. The relative expression level was calculated by the 2^−ΔΔCT^ method

Do the decreased GA content and increased ABA accumulation in *d3* and OE-miR528 lines result from the expression changes of key genes involved in GA and ABA biosynthesis and metabolism? To verify this, we analyzed the expression level of four GA degradation (*OsGA2ox1*, *OsGA2ox3*, *OsGA2ox4* and *OsEUI*), seven GA biosynthesis (*OsGA20x*, *OsGA3ox2*, *OsCPS1*, *OsCPS2*, *OsKS5*, *OsKO2* and *OsKAO*), three ABA oxidation (*ABA8OX1*, *ABA8ox2* and *ABA4*), and four ABA biosynthesis-related genes (*NECD1*, *NECD2*, *SDR1* and *AAO3*) in WT, OE-D3-9 and *d3-1* lines (Spielmeyer et al. 2002; Itoh et al. 2004; Oikawa et al. 2004; Sakamoto et al. 2004; Luo et al. 2006; Saika et al. 2007; Lo et al. 2008; Zhang et al. 2008, 2020c; Zhu et al. 2009; Toyomasu et al. 2009; Huang et al. 2010; Feng and Zhao 2011; Bang et al. 2013; Qin et al. 2013; Chi et al. 2014; Endo et al. 2014; Tong et al. 2014). The GA degradation-related genes *OsGA2ox3* and *OsEUI* were highly expressed in *d3-1* and OE-D3-9 lines, respectively (Fig. 6A). The expression of four GA biosynthesis-related key genes, including *OsCPS1*, *OsCPS2, OsKO2* and *OsKAO*, was significantly up-regulated in OE-D3-9 than that in WT and *d3-1* mutant (Fig. 6B). The expression level of the three ABA oxidation-related genes was similar in *d3-1* and OE-D3-9 lines (Fig. 6C). However, the ABA biosynthesis-related key gene *NECD2* was significantly up-regulated in *d3-1* plants (Fig. 6D). These results indicate that the changes of plant height of transgenic lines would be partially due to the expression changes of some GA and ABA biosynthesis and metabolism-related genes.

## Discussion

Plant height is one of the most important agronomic traits, which largely affects crop yields. During the past decades, a number of dwarf mutants have been identified in rice, of which *d3* is a tillering dwarf mutant caused by the mutation in an F-box protein with leucine-rich repeats (Ishikawa et al. 2005; Yan et al. 2007). In the present study, we newly generated two allelic mutants, *d3-1* and *d3-3*, through CRISPR/Cas9 system (Additional file 1: Fig. S2). Both the two homozygous mutants exhibit dwarf phenotype during the whole growth stages, which results from the abnormal elongation of internode cells (Figs. 2, 3). But, *d3-3* has a much severer tillering dwarf phenotype than *d3-1*, which may be due to the introduced loss-of-function mutation that caused its prematurely transcription termination. On the contrary, *D3*-overexpressing plants are relatively higher than WT (Fig. 2 and Additional file 1: Fig. S3). Accordingly, *D3* should be a crucial gene in regulation of plant height in rice.

miR528 is a monocot-specific miRNA, which plays multifunctional roles in plants (Wu et al. 2017; Tang and Thompson 2019; Yang et al. 2019; Zhang et al. 2020a). But, whether miR528 regulates plant height remains unknown. In rice, *D3* is one of the target genes of miR528 with the target site located in the 3’-UTR (Li et al. 2010; Zhou et al. 2010; Tang and Thompson 2019). We found that the plant height of OE-miR528 transgenics was significantly lower than WT, but was greatly higher than the *d3* mutants (Fig. 2 and Additional file 1: Fig. S3). Moreover, there were remarkably more number of tillers in OE-miR528 than WT, while the tiller number of OE-MIM528 was slightly fewer than WT plants (Additional file 1: Fig. S3). These observations indicate that in rice, miR528 plays an important role in regulation of both plant height and tiller number by targeting the *D3* gene. However, in OE-miR528 lines, the *D3* expression may maintain at a relative level, although it was substantially down-regulated by miR528, suggesting that the plant height may be closely positively related to the transcriptional abundance of *D3* gene in rice (Additional file 1: Fig. S1).

As reported, BRs play important roles in regulation of plant height (Hong et al. 2005; Zhang et al. 2016; Tong et al. 2018). However, are BRs involved in the regulation of plant height by the miR528-*D3* module? It was found that the contents of endogenous brassinolide (BL), castasterone (CS), and 6-Deoxocastasterone (6-DS) in OE-miR528-10 and OE-MIM528-1 lines were not significantly different from that of the WT plants (Additional file 1: Fig. S4A–C). SLs are endogenous phytohormones that regulate diverse physiological processes, including shoot branching and elongation (Sang et al. 2014). *D3* encodes an F-box containing protein, and is critical for SL signal transduction to suppress the shoot branching through assembled into an SCF complex and associated with D14 in rice (Zhao et al. 2014). It is therefore intriguing to further examine whether the change of *D3* expression will affect the SL accumulation in transgenic plants. We measured the endogenous 2’-*epi*-5-Deoxystrigol content in OE-miR528 and OE-MIM528 transgenic seedlings, and found that compared with WT, the content of 2’-*epi*-5-Deoxystrigol was significantly lower and higher in OE-miR528-10 and OE-MIM528-1 lines, respectively (Additional file 1: Fig. S4D).

Accumulating evidence shows that plant hormones have synergistic effects on each other. For instance, SL-deficient mutants *la1* and *d3* have increased IAA contents in both shoot base and whole seedlings, indicating that SL regulates the tiller/branch angle by inhibiting auxin biosynthesis in rice (Sang et al. 2014). The SL-insensitive (*d14*) and SL-deficient mutants (*d17*) are dwarf, and have reduced bioactive GA_1_ contents compared with WT, which could be rescued by application of exogenous GA_3_ (Zou et al. 2019). Accordingly, we speculate that D3 should be involved in multiple regulatory pathways in response to different plant hormones. In this study, the *d3* mutants have lower GA_4_ content and higher ABA content, which is more sensitive to GA but not to ABA; The dwarfness of *d3* mutants could be rescued by exogenous GA_3_ supplementation (Fig. 5). By contrast, OE-D3 seedlings have increased GA_4_ content and restored GA_3_ and ABA sensitivity like WT plants (Figs. 4, 5). In view of the antagonizing regulation of GA and ABA in plants, it is reasonable to infer that miR528-*D3* regulates shoot elongation by regulating the expression of the GA and ABA metabolic pathway-related genes (Fig. 6). Therefore, *D3*, as a key regulatory node, may control plant height by mediating the signaling and metabolism of SL, GA, and ABA phytohormones in rice.

## Conclusion

In the present study, the functional role of the miR528-*D3* module in regulation of plant height in rice was investigated. It was found that miR528 and its targeting gene *D3* were oppositely expressed in tested tissues along developmental stages, where miR528 was mainly expressed in roots and leaves at the maturity stage. The plant height of *d3* and OE-miR528 lines was significantly lower than WT, OE-D3, and OE-MIM528 lines, with the former transgenics having severely shortened internodes. However, both WT and transgenics had the same number of internodes, while the cell length of internodes of OE-D3 and OE-MIM528 lines was extensively longer than those of *d3* and OE-miR528 plants that contained apparently lower level of GA_4_ but higher ABA content. The supplementation of exogenous GA_3_ (1 µM) and ABA (3 µM) promoted the increase of plant height of *d3* and OE-miR528 lines, indicating that these transgenic lines were much more sensitive to external GA but not to ABA. Furthermore, GA biosynthesis-related genes (*OsCPS1*, *OsCPS2, OsKO2* and *OsKAO*) were strongly expressed in OE-D3 plants, while ABA biosynthesis-related gene *NECD2* was highly expressed in *d3* mutant. The results are useful for further understanding and manipulating the regulatory mechanism of plant height in rice.

## Materials and Methods

### Plant Materials

*Oryza sativa* L. *japonica* cv. Nipponbare was used as the wild type (WT) and genetic transformation in this study. The miR528 overexpressing plants (OE-miR528) and the miR528 target mimicry lines (OE-MIM528) were developed in our previous studies (Liu et al. 2015; Wang et al. 2021). The OE-miR528, OE-MIM528, OE-D3 lines, and *d3* mutants were created with the Nipponbare background and used in the following experiments.

### Generation of Transgenic Rice Plants

Using the full-length cDNA from the cv. Nipponbare as the template, the coding sequence (CDS) of the *D3* gene (LOC_Os06g06050) was amplified using the specific primers listed in Additional file 1: Table S1. The purified PCR product was then cloned into the *pCAMBIA1300-UBI-RBCS* vector using the Hieff Clone® Plus One Step Cloning Kit. The *D3* knockout vector was constructed using the CRISPR/Cas9 method. Based on the genomic sequence of *D3* gene, two specific CRISPR target sites were designed in the protein-encodable region. After determining the sites to be knocked out, the 23–25 bp exon sequence was selected, and subjected to obtain the sgRNA target site sequence for the specifically edited *D3* gene by using the Guide Design Resources (http://crispr.mit.edu/). Th gRNA vector was subsequently cut with *Bsa*I and connected to the small fragment annealed at the target site. Then PCR amplification was performed to obtain the gRNA expression box containing the target site. After that, the recovered and amplified target site fragments were digested with *Bsa*I, and multi-fragmented with the Cas9 vector digested with *Bsa*I. The constructed CRISPR vector was then transferred into *E. coli* competent cells for proliferation. The constructs were separately transferred into Nipponbare by Agrobacterium-mediated transformation method to generate transgenic lines (Tzfira and Citovsky 2006).

The genomic DNA was extracted from the leaves of transgenic seedlings using the CTAB method, and used for positive identification. For knockout transgenic plants, the target site-specific primers were used for PCR amplification and sequencing to detect the editing sites (Additional file 1: Table S1).

### Quantitative Real-Time PCR (qRT-PCR)

Total RNA was extracted from different tissues using TRIzol reagent (TaKaRa). After treated with DNase I, the RNA was used to synthesize cDNA using the Hifair™ III 1st Strand cDNA Synthesis Kit (YEASEN, China, http://www.yeasen.com). qRT-PCR was conducted in a BIO-RAD CFX96 Real-Time PCR System using the Hieff® qPCR SYBR® Green Master Mix (YEASEN, China, http://www.yeasen.com), according to the manufacturer’s instructions. The total volume of the reaction system was 20 μL, including Hieff® qPCR SYBR Green Master Mix, 10 μL, Forward primer, 0.4 μL, Reverse primer, 0.4 μL, Template of cDNA, 9 μL, and RNase-free water, 0.2 μL. The reaction procedure for qRT-PCR was as follows: 95℃ for 5 min, followed by 40 cycles of 95℃ for 10 s, 55–60℃ for 20 s, and 72℃ for 20 s. All experiments were performed with three biological repeats and three technical replicates. The *Ubquitin* gene was used as the internal control for normalization. The 2^−ΔΔCT^ method was adopted to evaluate the relative expression level of corresponding genes. Primers used for qRT-PCR were listed in Additional file 1: Table S2.

### Histological Analysis of Cell Morphology

To explore the cell morphology of WT and transgenic plants, the same region of the uppermost internode was collected from 31-day-old seedlings, and fixed in 75% alcohol solution immediately. The rice samples were heated in a water bath until the leaves turned into white completely, and then oven-dried at 37℃ overnight. The samples were put under an optical microscope to observe the cell morphology. The cell length of the uppermost internode was measured using ImageJ (Collins 2007). The measurement was conducted for more than 15 cells each of three plants per line.

### Determination of Endogenous Hormone Content

For GA_1_, GA_4_ and ABA quantification, 0.2 g of stems of 31-day-old WT and transgenic rice plants were collected and powdered with liquid nitrogen. Each sample was extracted in acetonitrile according to the method from Liu et al. (2020). For brassinolide (BL), castasterone (CS), and 6-Deoxocastasterone (6-DS) quantification, 2.0 g of stems of 31-day-old WT and transgenic rice plants were collected and extracted in 95% methanol, according to the previously described method (Kim et al. 2005). For the measurement of 2’-*epi*-5-Deoxystrigol content, 2.0 g of stems of 31-day-old WT and transgenic rice plants were collected and extracted in acetonitrile, using the method from Wang et al. (2020a, b). After extraction and purification, all samples were subjected to LC/MS–MS analyses using an Acquity Ultra Performance Liquid Chromatograph (LC-30AD, Shimadzu) coupled to a triple quadruple tandem mass spectrometer (SCIEX-6500Qtrap, SCIEX). Each assay was performed with three biological replicates.

### Exogenous GA and ABA Treatment

Rice seeds were sterilized with 0.5% NaClO solution for 15 min, and washed three times with sterile distilled water, and then immersed in water for two days in a constant temperature incubator at 32℃. Germinated seeds were grown on a nylon net floating on hydroponic solution (pH 5.8) (Shim et al. 2021). The 14-day-old seedlings of WT and transgenic lines were treated with 1 μM GA_3_ or 3 μM ABA for two weeks. Each treatment experiment was performed with three replicates with twelve seedlings. The nutrient solution was renewed every three days. After the 7 and 14 d of hormone treatment, the shoot length of each plant was measured (Chu et al. 2019; Chen et al. 2021).

## Supplementary Information

**Additional file 1.** **Fig. S1.** Expression analysis of the *D3* gene in **A** OE-MIM528, OE-miR528 and **B** OE-D3 transgenic plants. **Fig. S2.** Sequence analysis of *d3* mutants generated by CRISPR/Cas9. **A** The target sites and sanger sequence of *d3-1* and *d3-3* mutants. **B** Deduced amino acid sequence alignment of *d3* mutants. **Fig. S3.** Phenotypes of plant height of different transgenic lines at the maturity stage. Bars, 10 cm. **Fig. S4.** Comparison of the contents of endogenous brassinolide (BL) (**A**), castasterone (CS) (**B**), 6-Deoxocastasterone (6-DS) (**C**), and 2′-*epi*-5-Deoxystrigol (**D**) in 31-day-old OE-miR528 and OE-MIM528 transgenic seedlings. **Table S1.** Primers used for vector construction and positive identification. **Table S2.** Primers used for qRT-PCR analysis.

## Data Availability

The datasets supporting the conclusions of this article are included within the article and its additional files.

## Abbreviations

ABA: Abscisic acid
BR: Brassinosteroids
*D3*: Dwarf 3
GA: Gibberellin
IM: Intermediate meristem
miRNA: MicroRNA
SL: Strigolactone
SAM: Shoot apical meristem
WT: Wild type
