# Supplementary material for: The miR528-D3 Module Regulates Plant Height in Rice by Modulating the Gibberellin and Abscisic Acid Metabolisms
Source: Rice (N Y). 2022 May 20;15:27. doi: 10.1186/s12284-022-00575-3 (PMC9123139; doi:10.1186/s12284-022-00575-3)
Supplement: Supplementary file 1 — Additional file 1. Fig. S1. Expression analysis of the D3 gene in A OE-MIM528, OE-miR528 and B OE-D3 transgenic plants. Fig. S2. Sequence analysis of d3 mutants generated by CRISPR/Cas9. A The target sites and sanger sequence of d3-1 and d3-3 mutants. B Deduced amino acid sequence alignment of d3 mutants. Fig. S3. Phenotypes of plant height of different transgenic lines at the maturity stage. Bars, 10 cm. Fig. S4. Comparison of the contents of endogenous brassinolide (BL) (A), castasterone (CS) (B), 6-Deoxocastasterone (6-DS) (C), and 2′-epi-5-Deoxystrigol (D) in 31-day-old OE-miR528 and OE-MIM528 transgenic seedlings. Table S1. Primers used for vector construction and positive identification. Table S2. Primers used for qRT-PCR analysis. [file 12284_2022_575_MOESM1_ESM.pdf]

Supplementary Information

The miR528-*D3* module regulates plant height in rice by modulating the gibberellin and abscisic acid metabolisms

Juan Zhao[#], Xing Liu[#], Mei Wang, Lingjuan Xie, Zhengxin Wu, Jiuming Yu, Yuchen Wang, Zhiqiao Zhang, Yufang Jia, Qingpo Liu[*]

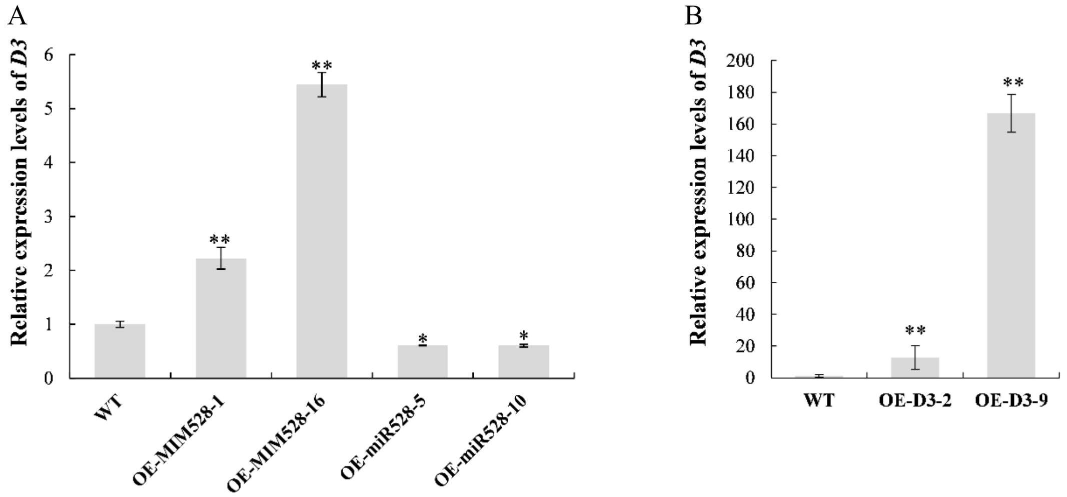

Figure S1. Expression analysis of the *D3* gene in (A) OE-MIM528, OE-miR528 and (B) OE-D3 transgenic plants.

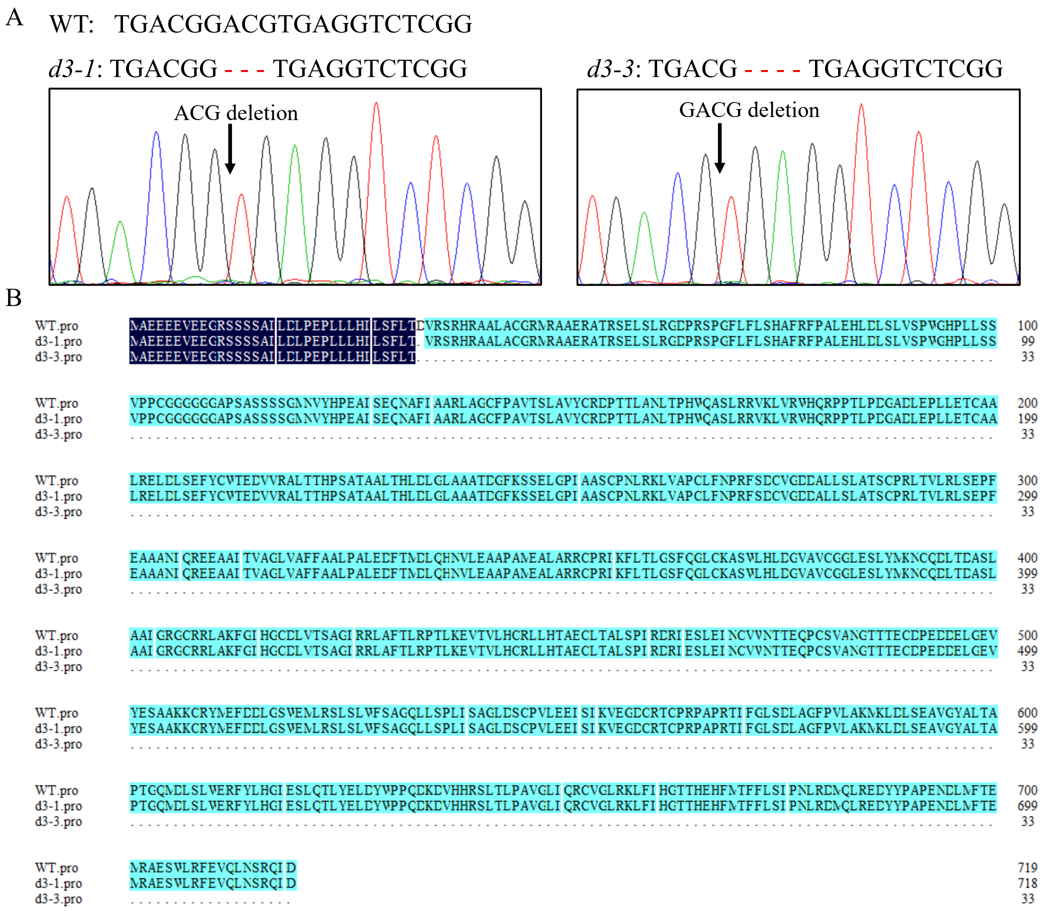

Figure S2. Sequence analysis of *d3* mutants generated by CRISPR/Cas9.

(A) The target sites and sanger sequence of *d3-1* and *d3-3* mutants. (B) Deduced amino acid sequence alignment of *d3* mutants.

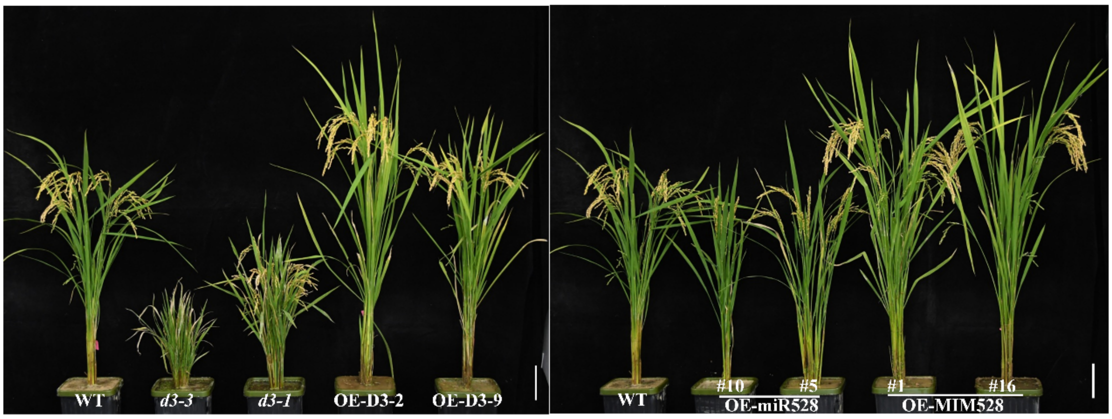

Figure S3. Phenotypes of plant height of different transgenic lines at the maturity stage. Bars, 10 cm.

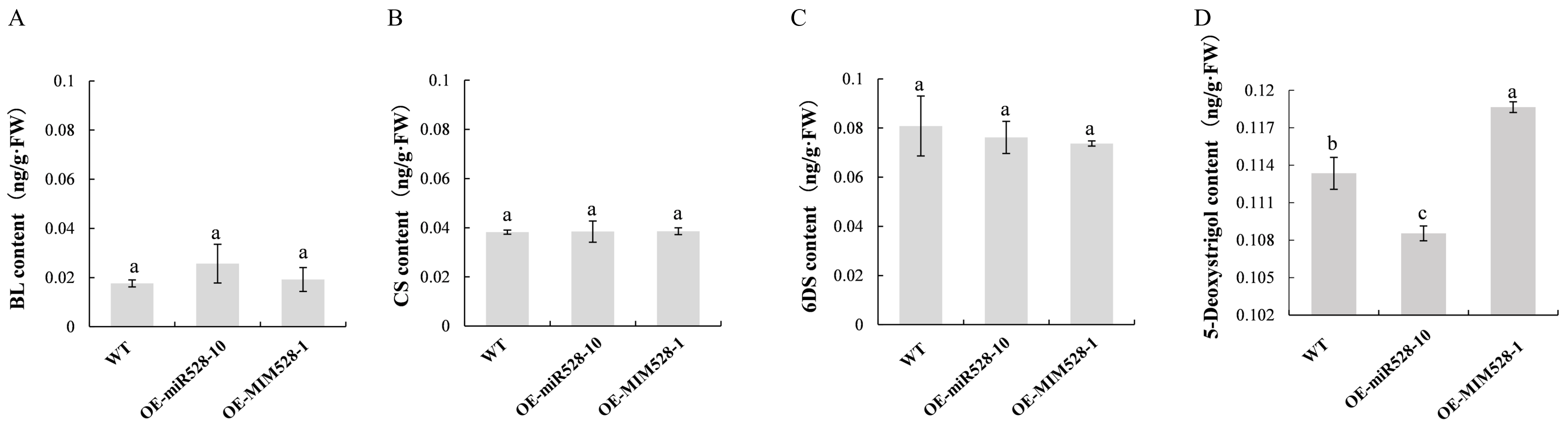

Figure S4. Comparison of the contents of endogenous brassinolide (BL) (A), castasterone (CS) (B), 6-Deoxocastasterone (6-DS) (C), and 5-Deoxystrigol (D) in 31-day-old OE-miR528 and OE-MIM528 transgenic seedlings.

Table S1. Primers used for vector construction and positive identification.

Primer	Sequence
D3cds-F	GCTGGATCCATGGCGGAAGAGGAGGAGGT
D3cds-R	GGACTAGTCCATAATCATAACCAACTCACCAGGA
U6aF	gccGCCGAGACCTCACGTCCGTC
U6aR	aaacGACGGACGTGAGGTCTCGG
U6bF	gttGGAAGCGGAACGCGTGCGAG
U6bR	aaacCTCGCACGCGTTCCGCTTC
SP-F	GCGCGGTGTCATCTATGTTACTA
SP-R	CCCGACATAGATGCAATAACTT
hpt557-F	ACACTACATGGCGTGATTTCAT
hpt557-R	TCCACTATCGGCGAGTACTTCT
d3-F	TTCACCCCAAATCCCTCAAC
d3-R	AGATCGCCTCGGGGTGGTAC

Note: D3cds-F/D3cds-R were used for cloning of *D3* gene; U6aF/U6aR, U6bF/U6bR were two specific CRISPR target sites for knockout of *D3* gene; SP-F/SP-R were used for identification of CRISPR/Cas9 tag; hpt557-F/hpt557-R were used for the identification of hygromycin; d3-F/d3-R were used for positive identification of transgenic seedlings.

Table S2. Primers used for qRT-PCR analysis

Gene	Primer	Sequence
OsGA20ox1	OsGA20ox1-FP	TACGCCAGCAGCTTCACGG
	OsGA20ox1-RP	TCCATCAGCTCCAGCGACA
OsGA20ox2	OsGA20ox2-FP	GCAACTACTACCCGCCAT
	OsGA20ox2-RP	CAGGCAGCTCTTATACCTCC
OsGA20ox3	OsGA20ox3-FP	CGCTCACCTTCTTCCTCAACC
	OsGA20ox3-RP	AGCCATTCCTTTGCTTGATCCA
OsGA20ox4	OsGA20ox4-FP	CGCTTCGTCGACAACCTC
	OsGA20ox4-RP	CTGTCCTCGAAGAACTCCC
OsGA3ox1	OsGA3ox1-FP	GATCTCTTCCATGTGCTCACC
	OsGA3ox1-RP	GAATCATGCTCAACGCCGAT
OsGA3ox2	OsGA3ox2-FP	TCTCCAAGCTCATGTGGTCCGAGGGCTA
	OsGA3ox2-RP	TGGAGCACGAAGGTGAAGAAGCCCGAGT
OsGA2ox1	OsGA2ox1-FP	CGAGCAAACGATGTGGAAGGGCTACAGG
	OsGA2ox1-FP	TGGCTCAGGCGGAGTGAGTACATTGTCG
OsGA2ox2	OsGA2ox2-FP	CCCCACATCCCTGACAAGGCTC
	OsGA2ox2-RP	CTATTCATGGTCGTCATCGTCC
OsGA2ox3	OsGA2ox3-FP	TGAGCGCGCTGGTGACGGCGGA
	OsGA2ox3-RP	CTTGATTTGTAGGCAGCCTTC
OsGA2ox4	OsGA2ox4-FP	TCGGTGGAGGATAACTTCGGC
	OsGA2ox4-RP	TGGGTTAGCGACAGGTGGTGG
OsGA2ox5	OsGA2ox5-FP	ATGGAGGAGCACGACTACGACT
	OsGA2ox5-RP	TCCTCCATGATCTGCTTCCTGTA
OsGA2ox6	OsGA2ox6-FP	GACGACGTGCTTCCTGCGGCTCAA
	OsGA2ox6-RP	CTTCCTGCACCTTCTTCCTGTA
OsGA2ox7	OsGA2ox7-FP	ACGGGAGCTTCTACGCGAGT
	OsGA2ox7-RP	TCAAATCTGCAGAGCCTGTCGTC
OsGA2ox8	OsGA2ox8-FP	GTGCTGCGGCGGATGGTGGTGG
	OsGA2ox8-RP	CTTCGTCGCGGCCTCATCGTTGG
OsGA2ox9	OsGA2ox9-FP	ATGTCGAGGCTGGCCAGGG
	OsGA2ox9-RP	CATACGAGGAAATTACTGAGGC
OsGA2ox10	OsGA2ox10-FP	ATGAGACAGCTCCGCCGTCTCTGG
	OsGA2ox10-RP	TTACGTCGTTGTGTTCGATCGTC
OsGA2ox11	OsGA2ox11-FP	CTCCGATCCAACGACACCTCT
	OsGA2ox11-RP	AGCCAGCGCCTCGTCCTGAT
OsCPS1	OsCPS1-FP	ACGAATTGAGGAGGCAGCATCTATG
	OsCPS1-RP	GAGCAAGTTCTTGCATACCCAACTC
OsCPS2	OsCPS2-FP	CTCTTCCATGTGCAGCAAAC
	OsCPS2-RP	CATGCTGGTAGACACAACTC
OsKS2	OsKS2-FP	AGATCGTCACCGAGCGCGACCTCG
	OsKS2-RP	TGACGGCAGGAACCTCCAAACCCATCTC
OsKS5	OsKS5-FP	CAAACTCCACGCTTCCCGCAATGTATCG
	OsKS5-RP	GAATTCCAAACCCGTTCCAGTGGCAAGG
OsKO2	OsKO2-FP	ATTTCTTCCCCTACCTCAGCTGGGTTCC
	OsKO2-RP	CTCTATGAGTGCCTCCCACACTAGCATC
OsKAO	OsKAO-FP	GAGATCGTCGACGTCCTCATCATGTACC
	OsKAO-RP	AGATGTTGACGCAGCGAAGTGTCTCGTC
EUI	EUI-FP	GCGTTGCCGGTGGTGGTGGC
	EUI-RP	GAGGGGTCTCCTCCCCGTC
